# Response of human melanoma cell lines to interferon-beta gene transfer mediated by a modified adenoviral vector

**DOI:** 10.1038/s41598-020-74826-y

**Published:** 2020-10-21

**Authors:** Taynah I. P. David, Otto L. D. Cerqueira, Marlous G. Lana, Ruan F. V. Medrano, Aline Hunger, Bryan E. Strauss

**Affiliations:** 1 Laboratório de Vetores Virais, Centro de Investigação Translacional em Oncologia, Instituto Do Câncer Do Estado de São Paulo, Faculdade de Medicina, Universidade de São Paulo, Av. Dr. Arnaldo, 251, 8th floor, São Paulo, SP Brazil; 2 Present Address: Department of Pathology and Immunology, Washington University School of Medicine, St. Louis, MO USA; 3 Present Address: Cristalia, Biotecnologia Unidade 1, Rodoviária SP 147, Itapira, SP Brazil

**Keywords:** Cancer therapy, Cell death

## Abstract

Since melanomas often retain wild type p53, we developed an adenoviral vector, AdRGD-PG, which provides robust transduction and transgene expression in response to p53. Previously, this vector was used for interferon-β gene transfer in mouse models of melanoma, resulting in control of tumor progression, but limited cell killing. Here, the AdRGD-PG-hIFNβ vector encoding the human interferon-β cDNA (hIFNβ) was used to transduce human melanoma cell lines SK-MEL-05 and SK-MEL-147 (both wild type p53). In vitro, cell death was induced in more than 80% of the cells and correlated with elevated annexinV staining and caspase 3/7 activity. Treatment with hIFNβ promoted cell killing in neighboring, non-transduced cells, thus revealing a bystander effect. In situ gene therapy resulted in complete inhibition of tumor progression for SK-MEL-147 when using nude mice with no evidence of hepatotoxicity. However, the response in Nod-Scid mice was less robust. For SK-MEL-05, tumor inhibition was similar in nude and Nod-Scid mice and was less efficient than seen for SK-MEL-147, indicating both cell type and host specific responses. The AdRGD-PG-hIFNβ vector provides extensive killing of human melanoma cells in vitro and a potent anti-tumor effect in vivo. This study provides a critical advance in the development of our melanoma gene therapy approach.

## Introduction

Melanoma is known for its high lethality^1^. If detected early, while still a localized tumor, surgical removal is often curative^2^. However, after metastatic dissemination, prognosis worsens and the average life expectancy is no more than 1 year^3^. Despite many advances in therapy over the past decade, many of these patients remain refractory to treatment and available options for intervention are not effective^4–7^. However, the genotype of melanomas may offer targets for its treatment.

While 80% of melanomas preserve p53 in its wild type form^8^, deletions are commonly found in the chromosome 9p21 gene cluster, where CDKN2a, p14ARF and interferon-β (IFNβ) are located^9–12^. The IFNβ protein is a type I interferon, a family of cytokines that possess pleiotropic actions, including modulation of innate and adaptive immune responses. Upon binding of IFNα/β to the interferon alpha receptor (IFNAR), signal transduction mediated by the Jak-STAT system then activates the interferon stimulated gene factor 3 (ISGF3) complex, which in turn translocates to the nucleus and promotes transcription of a range of genes containing the interferon-sensitive response element (ISRE) in their promoters. Studies also support the notion that loss of the genes encoding IFNα/β is a frequent event in carcinogenesis contributing to an immunosuppressive microenvironment^13,14^. For metastatic melanoma, the IFNA1/IFNB1 locus is altered in 11% of cases (www.cbioportal.org). Thus, the therapeutic use of type I interferon as an antitumor agent is justified.

In fact, recombinant IFNβ protein is used to treat some forms of cancer, although its efficacy may be limited. The systemic administration of cytokines requires high dosages due to the short half-life of the protein, which besides being nonspecifically distributed, generates side effects and toxicities^15–18^. Alternatively, intratumoral gene therapy using viral vectors encoding the IFNβ cDNA should provide localized, yet high level expression. Clinical studies have shown some benefit when using IFNβ gene therapy for the treatment of glioma, retinoblastoma, melanoma and mesothelioma^19–22^. However, improved vector design may enhance the efficiency of viral transduction and expression of the therapeutic gene.

In our previous work, we have shown that an adenoviral vector with two key modifications provides superior performance in models of cancer gene therapy. Specifically, we have incorporated the RGD-modified fiber as well as a p53-responsive promoter, termed PG, to drive transgene expression. The RGD (arginylglycyl aspartic acid) modification broadens the tropism of the virus since it no longer relies on the native adenovirus receptor and now interacts with integrins which are widely encountered on the surface of many cell types^23^. The PG promoter provides high levels of expression in response to wild-type p53 and may be used to drive the expression of p53 itself or other transgenes of interest^24–27^. Thus, the vector employed here provides enhanced transduction and transgene expression^24,25^. The AdRGD-PG vectors have been used for the transfer of IFNβ to mouse models of melanoma^24,28,29^ and lung carcinoma^30^ that harbor wild-type p53, resulting in reduced tumor progression, but limited cell killing. However, the performance of the AdRGD-PG vector in human melanoma cells is just beginning to be explored.

Here we show that transfer of human IFNβ mediated by the AdRGD-PG vector results in extensive cell death and provides a desirable bystander effect in vitro for both the SK-MEL-05 and SK-MEL-147 cell lines. In situ gene therapy resulted in nearly complete eradication of SK-MEL-147 tumors in nude mice. With this study, we have shown that the improved vector confers high level cell killing and effective control over tumor progression upon transfer of human IFNβ.

## Results

### Adenovirus carrying hIFNβ induces cell death in human melanoma cell lines

**Figure 1:**
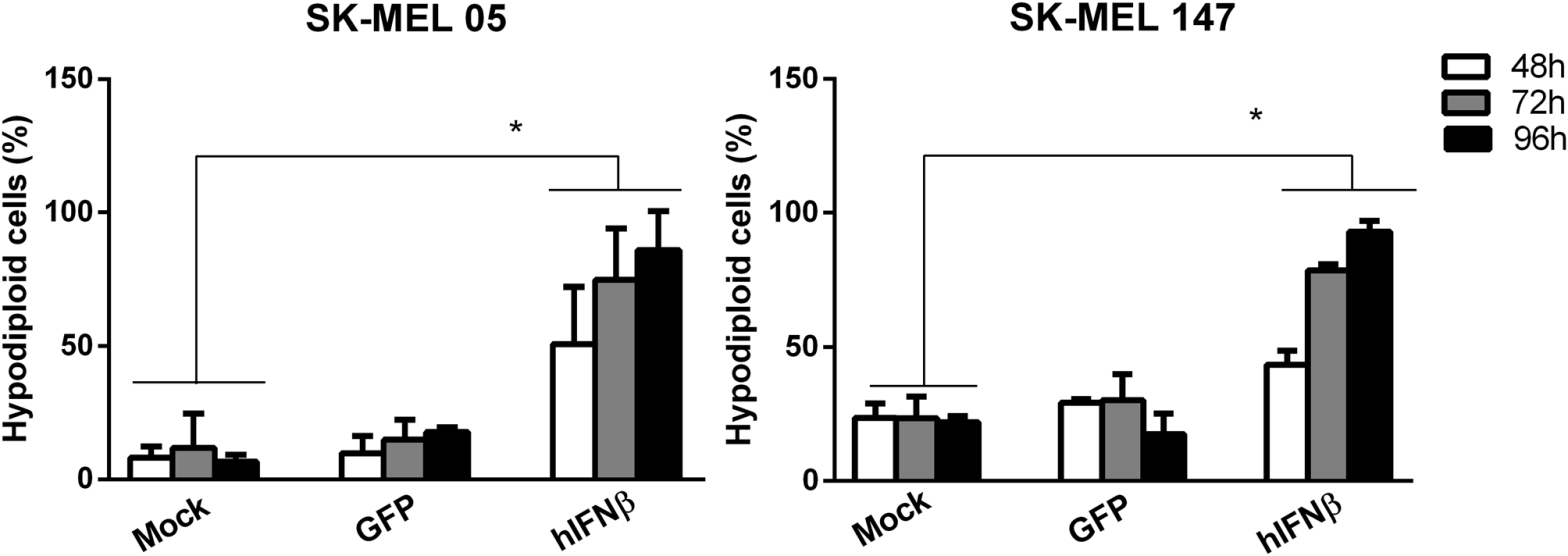
Transduction of human melanoma cell lines with an adenovirus carrying hIFNβ induces the accumulation of a hypodiploid cell population. SK-MEL-05 and SK-MEL-147 cells were transduced with AdRGD-PG-eGFP (GFP) or AdRGD-PG-hIFNβ (hIFNβ) using an MOI of 100 or non-treated cells (Mock) were used as a control. Determination of cells containing fragmented DNA was performed using propidium iodide labeling 48, 72 and 96 h after transduction. Graphs depict the mean and standard deviation from three independent assays each performed with technical duplicates. Statistical analysis was performed using the One-way ANOVA test followed by the Tukey’s range test using GraphPad Prism version 5.0 for Windows (GraphPad Software, La Jolla California USA). *p < 0.05.

In this work, we used the AdRGD-PG vector for the transfer of the hIFNβ and eGFP cDNAs (Figure S1) to human melanoma cell lines SK-MEL-05 and SK-MEL-147 (both wild type p53^31,32^). Transgene expression, eGFP (Figure S2) or hIFNβ (Figure S3), was detected. To investigate the potential of the AdRGD-PG-hIFNβ vector to trigger cell death, the cell lines were transduced and, after 48, 72 or 96 h incubation, the hypodiploid cell population (containing fragmented DNA) was analyzed by flow cytometry. Figure 1 shows that AdRGD-PG-IFNβ induced cell death in greater than 80% of cells by 96 h in both cell lines (representative flow cytometry data presented in Figure S4).

### Characterization of cell death in response to hIFNβ gene transfer

**Figure 2:**
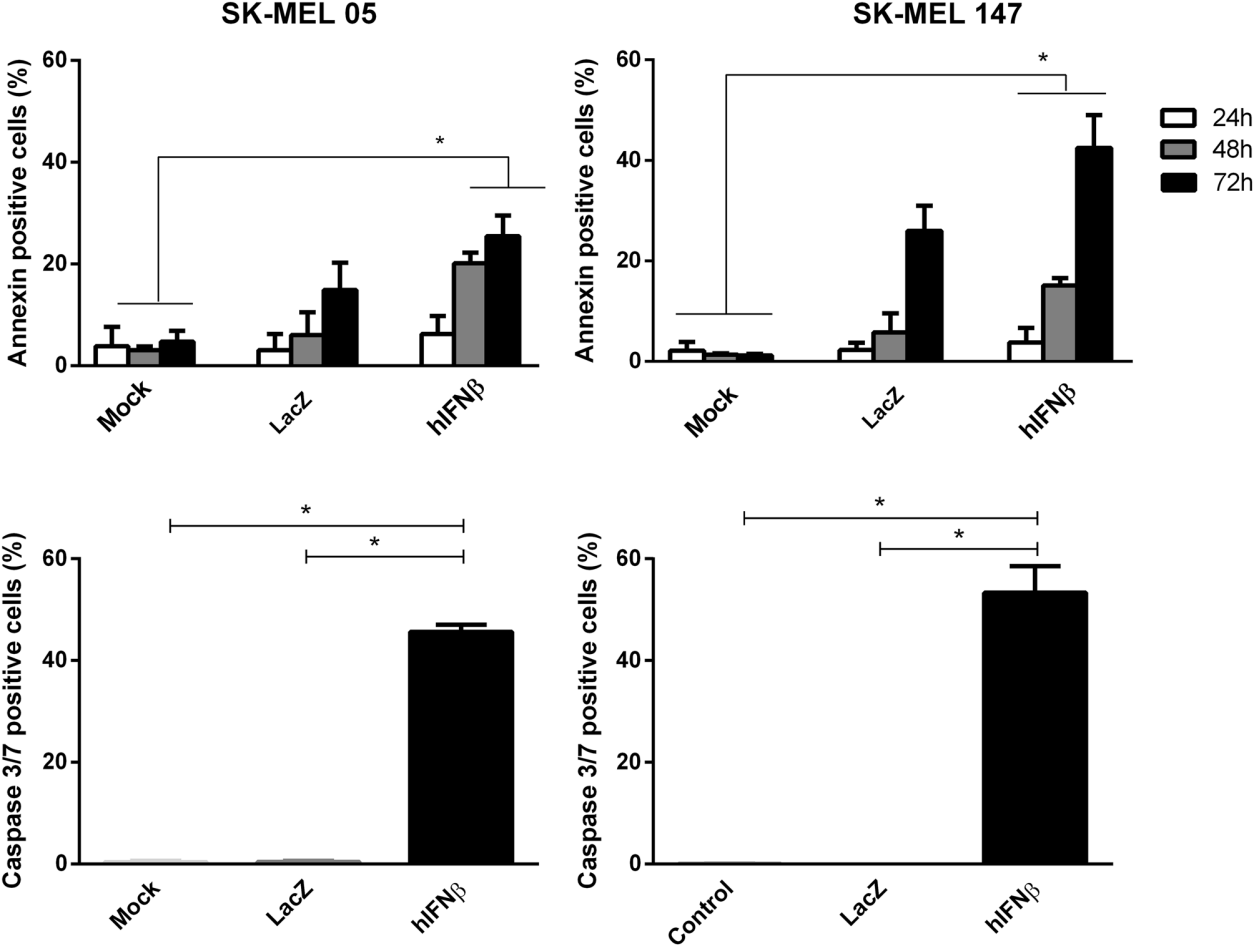
Characterization of cell death in response to adenoviral hIFNβ gene transfer. SK-MEL-05 and SK-MEL-147 cells were transduced with AdRGD-CMV-LacZ (LacZ) or AdRGD-PG-hIFNβ (hIFNβ) using an MOI of 100 or non-treated cells (Mock) were used as a control. In the upper panel, after 24, 48 or 72 h incubation, cells were harvested and stained with annexinV/PI. Graphs show percentage of the total cell population staining positive for annexinV + annexinV/PI. Alternatively, in the lower panel, the cells were tested for caspase 3/7 activity after 60 h incubation. In either case, graphs depict the mean and standard deviation from three independent assays each performed with technical duplicates. Statistical analyses were performed by the One-way ANOVA test followed by the Tukey’s range test using GraphPad Prism version 5.0 for Windows (GraphPad Software, La Jolla California USA). * *p* < 0.01.

In order to investigate the mechanism of cell death, the cell lines were transduced with AdRGD-PG-hIFNβ before annexin V/PI staining. In Fig. 2, we demonstrate that phosphatidylserine exposure increased in proportion with the time of incubation post-transduction (representative flow cytometry data presented in Figure S5). Caspase 3/7 activity was also elevated, seen in over 40% of cells, 48 h post-transduction with the adenoviral vector encoding hIFNβ (Fig. 2). The control vector, AdRGD-CMV-LacZ, did not provoke significant levels of annexin V staining at the 24 and 48 h time points, yet did at the 72 h time point. However, no caspase 3/7 activity was seen with this control (60 h time point). Thus, while the AdRGD-CMV-LacZ vector may have impacted exposure of phosphatidylserine, it did not induce cell death. For both the SK-MEL-05 and SK-MEL-147 lines, cell death triggered by the AdRGD-PG-hIFNβ vector is occurring by a mechanism consistent with apoptosis.

### hIFNβ secreted by transduced cells provides a bystander effect

**Figure 3:**
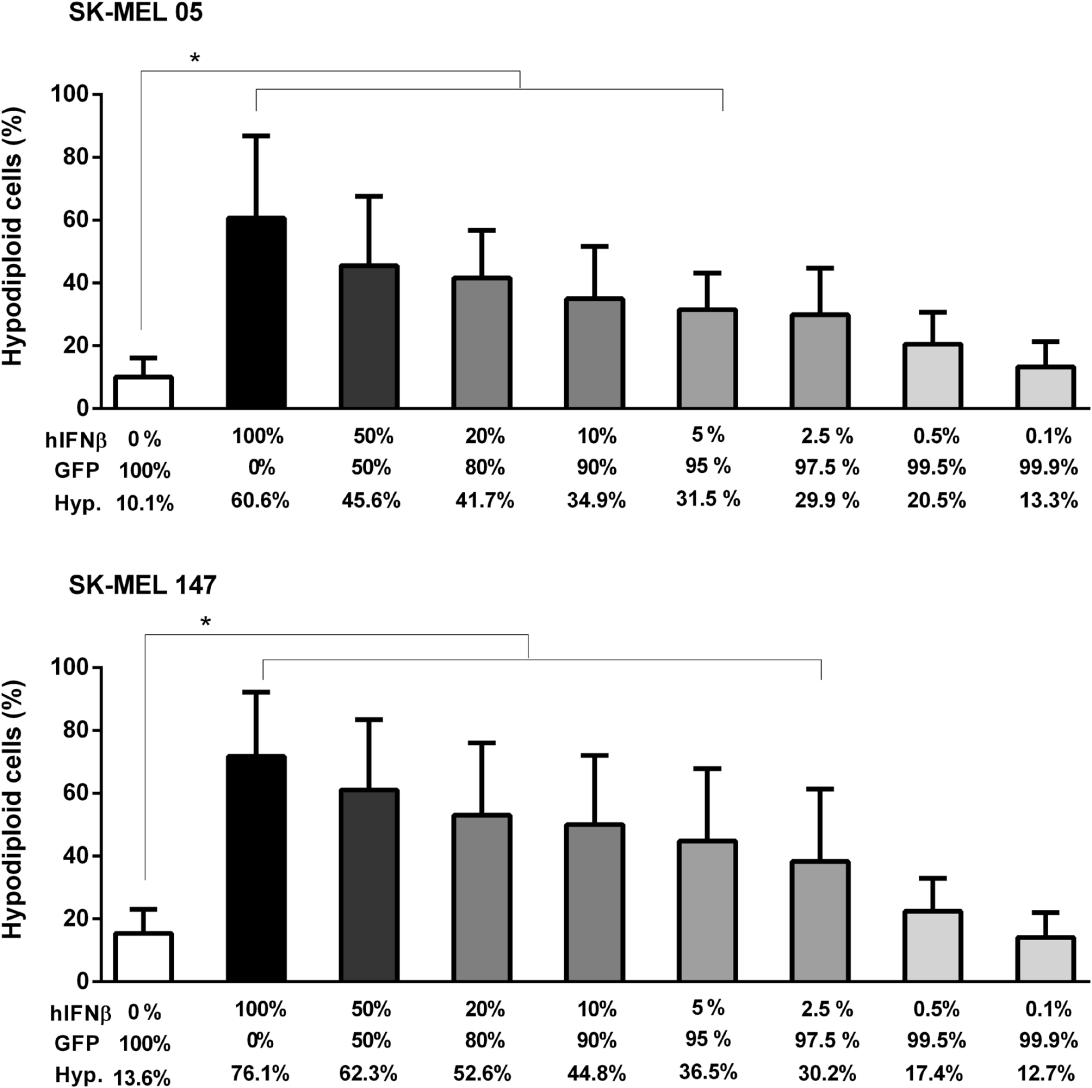
Bystander effect mediated by secreted IFNβ. Cells were transduced with AdRGD-PG-hIFNβ (hIFNβ) or AdRGD-PG-eGFP (GFP) and, the next day, harvested, counted and mixed at the indicated proportions before co-cultivation for 48 h before observation of the hypodiploid cell populations. Also indicated is the percentage of hypodiploid cells observed for each condition. Graphs depict the mean and standard deviation from three independent assays each performed with technical duplicates. Statistical analysis was performed by the One-way ANOVA test and followed by the Tukey’s range test using GraphPad Prism version 5.0 for Windows (GraphPad Software, La Jolla California USA). * *p* < 0.05.

Since hIFNβ is a secreted protein, we tested for a possible bystander effect on cells adjacent to those transduced with AdRGD-PG-hIFNβ. For this, cells transduced with AdRGD-PG-hIFNβ were co-cultured for 48 h in different proportions with cells transduced with the AdRGD-PG-eGFP control vector before evaluation of the hypodiploid population. In the case of SK-MEL-05, 5% of cells treated with AdRGD-PG-hIFNβ was sufficient to confer 40% hypodiploid cells, while 100% treated cells was correlated with 50% of cells harboring fragmented DNA (Fig. 3). The SK-MEL-147 cell line, on the other hand, showed greater sensitivity to the bystander effect, with transduction of 2.5% of cells with AdRGD-PG-hIFNβ being able to induce hypodiploidy in 40% of cells (Fig. 3). Thus, the bystander effect provides the benefit of hIFNβ gene transfer even to neighboring, non-transduced SK-MEL05 and SK-MEL-147 melanoma cells.

### In situ gene therapy with AdRGD-PG-hIFNβ confers potent inhibition of tumor progression

**Figure 4:**
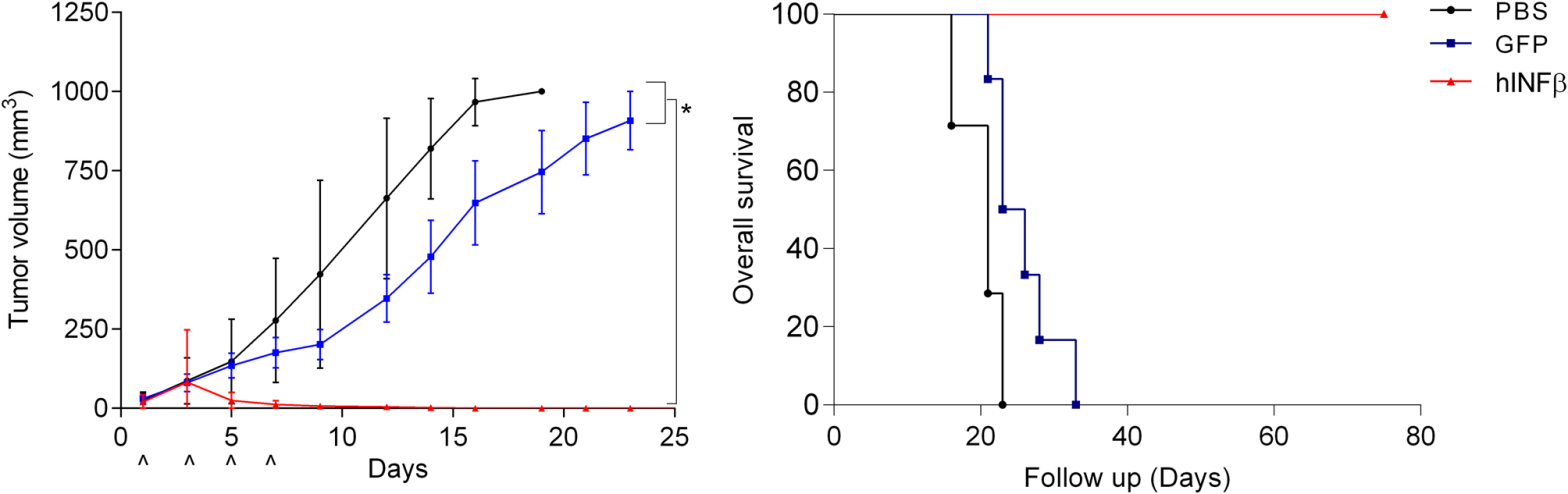
Xenograft model of in situ hIFNβ gene therapy shows elimination of tumor progression. Subcutaneous SK-MEL-147 tumors were established in female athymic nude mice before receiving a total of 4 intratumoral injections (as indicated in figure) with 2 × 10^8^ TU of AdRGD-PG-hIFNβ (IFNβ, n = 7), AdRGD-PG-eGFP (GFP, n = 7) or, as a control, PBS (n = 8). The graph shows tumor volume and arrows indicate the moment of treatment. Two-way ANOVA followed by Bonferroni post-test, * *p* < 0.05. Kaplan Meyer graph depicts overall survival (bearing sub-maximum tumor volume). Log-rank (Mantel-Cox) Test using GraphPad Prism version 5.0 for Windows (GraphPad Software, La Jolla California USA). *p* < 0.0001.

For the initial in vivo assay, SK-MEL-147 tumors were established s.c. in nude mice before receiving a total of four gene therapy applications at 48 h intervals, each using 2 × 10^8^ transducing units (TU). In Fig. 4, AdRGD-PG-hIFNβ conferred a statistically significant reduction in tumor volume and increase in overall survival compared to the mock and AdRGD-PG-eGFP controls. At the end of the observation period, 90 days after initiating treatment, none of the animals treated with hIFNβ gene therapy presented tumors, yet all of the animals treated with PBS or the eGFP control had reached maximum tumor volume by day 19 or 23, respectively. In parallel, a sub-set of these animals were euthanized 48 h after the final viral application (day 9) and serum from these animals was further analyzed. We detected the presence of hIFNβ only in serum of Ad-RGD-PG-hIFNβ treated animals (animal hIFNβ-1 and animal hIFNβ-2, 4006.8 and 1538.2 pg/ml, respectively). In addition, we investigated the occurrence of possible hepatotoxic effects resulting from this therapeutic regimen in the animals. ALT and AST assays did not show changes indicative of hepatic damage due to treatment (Figure S6).

**Figure 5:**
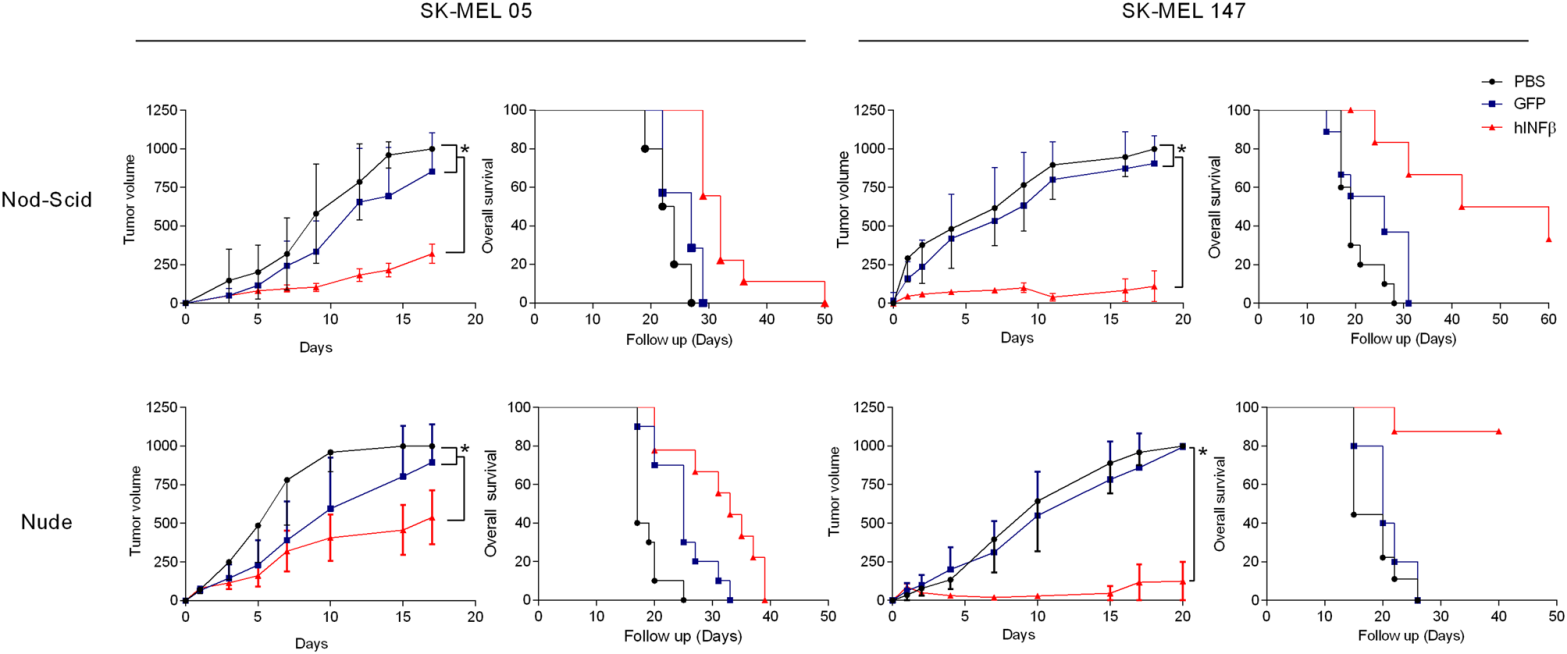
Cell type and model specific responses to in situ hIFNβ gene therapy. Human melanoma cell lines SK-MEL-147 and SK-MEL-05 were inoculated on the back of immunocompromised mice (Nude or Nod-Scid). When tumors reached 60 mm^3^, the therapeutic regimen was initiated, consisting of 4 intratumoral doses of 2 × 10^8^ TU of AdRGD-PG-hIFNβ (IFNβ, for SK-Mel-05, Nod-Scid n = 9, Nude n = 9; for SK-Mel-147, Nod-Scid n = 6, Nude n = 7), AdRGD-PG-eGFP (GFP, for SK-Mel-05, Nod-Scid n = 7, Nude n = 10; for SK-Mel-147, Nod-Scid n = 10, Nude n = 10) or, as a control, PBS (for SK-Mel-05, Nod-Scid n = 10, Nude n = 10; for SK-Mel-147, Nod-Scid n = 10, Nude n = 10). Tumor growth (left) and Kaplan–Meier (right) curves showing overall survival (sub-maximum tumor volume). Statistical analysis for the growth curve was performed using Two-way ANOVA followed by Bonferroni post-test, *p* < 0.0001, and for KM plots Log-rank (Mantel-Cox) Test, *p* < 0.0001 (GraphPad Prism version 5.0 for Windows, GraphPad Software, La Jolla California USA).

Encouraged by these results, we next evaluated the impact of gene therapy when applied to either the SK-MEL-05 or SK-MEL-147 tumors and using both nude and Nod-Scid mice as hosts (Fig. 5). For SK-MEL-05, tumor inhibition was substantial, though not as long lasting as seen for SK-MEL-147. Interestingly, the Nod-Scid host strain used in the xenograft model resulted in reduced survival when SK-MEL-147 cells were treated with hIFNβ as compared to the assay in nude mice, though this difference was not clearly seen in the case of SK-MEL-05. Consistent with SK-MEL-147 being generally more sensitive to hIFNβ gene transfer than SK-MEL-05, inhibition of tumor progression was more pronounced in the nude mouse model in the case of SK-MEL-147.

## Discussion

Here we have shown that the AdRGD-PG vector effectively inhibits tumor activity upon transfer of the human IFNβ cDNA. In vitro, both the SK-MEL-05 and SK-MEL-147 human melanoma cell lines were killed in the presence of exogenous hIFNβ, whether by transduction or through a bystander effect. The observation of annexinV staining and caspase 3/7 activity suggests a mechanism of cell death consistent with apoptosis. In situ gene therapy of the SK-MEL-147 cell line resulted in essentially complete abolition of tumor progression with no signs of toxicity when performed in nude mice. However, the same experiment performed in Nod-Scid mice was slightly less effective. While in situ gene therapy of SK-MEL-05 was similar in nude and Nod-Scid mice, the treatment was not completely effective. The in vivo assays suggest that differences in the cell type and in the host models may influence response to treatment.

The AdRGD-PG vector was developed in our prior studies in order to enhance both transduction and gene expression^24–26^. Moreover, we have shown in mouse models of melanoma and lung carcinoma that this vector may be used for the transfer of p19Arf plus hIFNβ in order to bring about high levels of cell death associated with the liberation of immunogenic factors and induction of an anti-tumor immune response^24,29,30^. In other words, we have used mouse models to demonstrate the efficacy of our immunotherapy based on gene transfer. Study of this approach in human cells is at its initial stages, but the work presented here represents a critical advance. As the literature shows, mouse models do not fully predict cellular response to human IFNβ^33^, emphasizing the importance of examining the response in human cells.

IFNβ was initially identified as an antiviral agent^34^. Subsequently, its actions on cell cycle arrest and induction of cell death were reported, including the demonstration that IFNβ treatment leads to the disruption of the mitochondrial membrane as well as activation of caspases associated with the mitochondrial and non-mitochondrial apoptosis pathways^35,36^. In agreement with these reports, our results show that transduction with AdRGD-PG-hIFNβ results in the accumulation of hypodiploid SK-MEL-147 and SK-MEL-05 populations and correlates with annexinV staining as well as caspase 3/7 activity. While this result was predicted, we point out that the cell killing was more efficient than that seen with gene transfer of IFNβ to mouse cancer cell lines in our previous work^24,29,30^, an important finding for our gene transfer approach.

Here, cell killing was evident even when only a portion of the cultured cells had been transduced, suggesting that secreted hIFNβ, and possibly additional factors, induced the death of the surrounding cells. However, IFNβ biochemotherapies are unsuccessful because of the short half-life of the recombinant protein and the patient's maximum tolerated dose. Alternatively, gene therapy with adenoviral vectors would be expected to provide high levels of localized IFNβ and, as a consequence, activation of interferon stimulated genes^37,38^. In gliomas IFNβ gene therapy has already been tested and demonstrated a clear inhibitory effect in some patients^19^. Perhaps further improvement of vector design would bring added efficacy to the gene therapy approach.

Application of AdRGD-PG-hIFNβ for in situ gene therapy of SK-MEL-147 tumors in nude mice resulted in essentially complete tumor inhibition while avoiding induction of hepatotoxicity as determined by serum ALT and AST levels. Interestingly, tumor inhibition was less effective when the assay was performed in Nod-Scid mice. Since a critical difference between these models is the presence of innate immunity in the nude mice^39–42^, we speculate that this may have contributed to the control of tumor progression. While in Nod-Scid mice, the lack of innate immunity may have contributed to tumor progression. In situ gene therapy using the SK-MEL-05 cell line performed similarly in either nude or Nod-Scid mice, suggesting that differences in the host strain were less critical than seen for SK-MEL-147. In addition, SK-MEL-05 was less responsive to AdRGD-PG-hIFNβ gene therapy, indicating a cell specific difference in efficacy.

Based on the findings shown here, our future endeavors will include several key investigations. Since IFNβ is an important immune modulator^43^, it will be of great interest to use appropriate models that reveal activation of distinct components of the innate and adaptive responses. In addition, previous demonstrations of our immunotherapy approach in mouse models required the transfer of both p19Arf and IFNβ^24,29,30^. Based on these findings, the inclusion of human p14ARF may provide additional control over tumor progression and, as indicated in our previous work, may contribute to the induction of immunogenic cell death. Another area of interest is combination therapies, such as gene transfer along with chemotherapy or checkpoint blockade.

We have shown that the AdRGD-PG vector is an effective means of transferring the human IFNβ cDNA to human melanoma cell lines, resulting in extensive cell death in vitro and nearly complete tumor inhibition when applied in a model of in situ gene therapy using SK-MEL-147 cells. While much work remains to be done, we propose that this gene transfer strategy may be further developed into an effective anti-cancer therapy.

## Methods

### Cell lines

SK-MEL 05 and SK-MEL-147 human melanoma cell lines (both wild type p53^31,32^) were authenticated by analysis of short tandem repeats, GenePrint 10 (Promega Internal, Standard-ILS 600, performed by the Rede Premium Core Facility, FMUSP) and tested negative for mycoplasma by a PCR assay using conditioned medium as template and amplification using the following oligonucleotides:

- Myco F: 5′-GGG AGC AAA CAC GAT TAG ATA CCC T-3′.
- Myco R: 5′-TGC ATT ATC TGT CAC TCT GTT AAC CTC-3′.

These cell lines as well as HEK293 were cultivated in DMEM with 10% fetal calf serum, supplemented with antibiotic–antimycotic (Thermo Fisher Scientific, Waltham, MA, USA) and maintained at 37 °C and 5% CO_2_ atmosphere.

### Construction, production, and titration of adenoviral vectors

The strategy for constructing the adenoviral vectors has been described previously^24^ and involves site specific recombination between the ‘entry’ vector encoding the gene of interest and the ‘destiny’ vector encoding the Ad5 backbone (non-replicating due to the deletion of E1, increased payload capacity due to the deletion of E3, and broad tropism/efficient transduction due to the inclusion of the RGD modified fiber which targets integrins). The cDNA for human interferon-β (hIFNβ) was isolated from plasmid pLG104R (ATCC 31,902) by the PCR method using the following oligonucleotides:

- huIFNβ-BamHI-F: 5′-CGT GGA TCC AAC ATG ACC AAC AAG TGT C-3.
- huIFNβ-BglII-R: 5′-TAG GAG ATC TTC AGT TTC GGA GGT-3′.

After amplification and cloning in pEntr-PG, site specific recombination was performed using pAdRGD-promoterless-Dest in the presence of LR Clonase (Thermo Fisher Scientific) following the manufacturer's recommendations. The construction of AdRGD-PG-eGFP (eGFP, enhanced green fluorescent protein) has described previously^24^. The AdRGD-CMV-LacZ vector was kindly provided by Dr. Hiroyuki Mizuguchi (Osaka University, Japan). Following viral amplification, purification was performed using an iodixanol gradient followed by desalting, as described by Peng et al.^44^ and as per our previous studies^24,25^. For the determination of biological titer, we used the Adeno-X Rapid Titer Kit (Clontech, Mountain View, CA, USA) which is based on immunodetection of the adenoviral hexon protein in transduced cells. The biological titer (particles capable of transducing a target cell, notated as Transducing Units per milliliter, TU/ml) was used for the calculation of the multiplicity of infection (MOI) indicated in each experiment.

### Evaluation of cell death by flow cytometry

Cells were plated, 5 × 10^4^ cells/well in 6 well dishes, and transduced the following day using an MOI of 100. Transduced cells were incubated for 48, 72 or 96 h then both detached and adherent cells were harvested, washed in 1 × PBS and fixed with 70% ethanol. Next, cells were treated with RNAse and stained with propidium iodide (PI) before samples were subjected to flow cytometry and analysis using the manufacturer's software (Attune and NxT Flow Cytometer Software, Thermo Fisher Scientific). The accumulation of hypodiploid (sub-G1) cells was quantified and interpreted as cell death.

### Annexin V / propidium iodide assay

SK-MEL-05 and SK-MEL-147 cells were transduced with the AdRGD-PG-hIFNβ and AdRGD-CMV-LacZ (used in place of the eGFP-encoding vector in order to avoid interference with FITC) vectors using an MOI of 100, and collected after 24, 48 and 72 h incubation. Dead Cell Apoptosis kit with annexin V FITC (Thermo Fisher Scientific, # V13242) and propidium iodide were used to stain these cells following the manufacturer’s recommendations. Briefly, after washing in 1 × PBS, the cells were suspended in Annexin Binding Buffer (10 mM HEPES, 140 mM NaCl and 2.5 mM CaCl_2_, pH 7.4) and labeled with Annexin V and PI (10 mg / ml) for 10 min at room temperature, protected from light exposure. Cytometry and the resulting analyses were performed using the Attune instrument and NxT Flow Cytometry Software (Thermo Fisher Scientific).

### Caspase activity assays

SK-MEL-05 and SK-5MEL147 cells were transduced with AdRGD-PG-hIFNβ using an MOI of 100. After 60 h incubation, the adherent and floating cells were collected and treated with Cell Event Caspase 3/7 Green Detection reagent (Thermo Fisher Scientific, Cat. No. C10423) diluted and incubated according to the recommendations of the manufacturer. The proportion of fluorescent cells resulting from activation of caspases 3 and 7 was assessed by flow cytometry (Attune, Thermo Fisher Scientific).

### Co-culture assays: determination of bystander effect

To explore a possible bystander effect, cells transduced with AdRGD-PG-hIFNβ were co-cultured with cells transduced with AdRGD-PG-eGFP at varying proportions before analyzing cell death. For this, SK-MEL-05 cells were transduced separately with either adenoviral vector at an MOI of 100 then, 24 h later, cells were trypsinized, washed and counted before admixtures (as indicated) were created, then plated and incubated for 48 h at which time cell death (hypodiploid cell population) was evaluated. The same was also performed using the SK-MEL-147 cell line.

### Mouse model of in situ gene therapy

Assays were performed using both athymic nude BALB/c or Nod-Scid mice in order to compare residual innate immunity^45^. In either case, 8–12 weeks old female mice were obtained from the Bioterio Central, FMUSP. SK-MEL-05 and SK-MEL-147 cells (1 × 10^6^) were implanted subcutaneously in the left flank and animals were observed until tumors reached 60 mm^3^ and treatment was initiated. Intratumoral injection was performed on four occasions at 48 h intervals (days 1, 3, 5, 7), applying 2 × 10^8^ TU in 50 µl of 1 × PBS as excipient. Tumors were treated with PBS, AdRGD-PG-eGFP or AdRGD-PG-hIFNβ, number of animals/group indicated in figure legends. Tumor width and length were recorded on alternate days, during and after treatment, for the calculation of tumor volume using the formula ½ × (length) × (width)^2^ ^46,47^. The experimental endpoint was 1000 mm^3^, at which time animals were euthanized in a chamber with 4% isoflurane, followed by CO_2_ inhalation. Alternatively, on experimental day 9 (48 h after the last treatment), some of these animals were euthanized and samples collected for histologic and/or serologic analyses. Animal experimentation was performed at the Centro de Medicina Nuclear (CMN), Faculdade de Medicina, Universidade de São Paulo (FMUSP). All experimental protocols were carried out in accordance with relevant guidelines and regulations and were approved by the Ethics in Animal Use Committee (CEUA), FMUSP, protocol number 016/11. While there was no predetermined selection of which animals would be included in each experimental condition, the distribution of animals/cage was maintained.

### Detection of IFNβ protein

The production of IFNβ was detected by ELISA (Verikine Human Interferon-β ELISA kit, PBL Assay Science, Piscataway, NJ, USA). For the in vitro assay, cells were plated, transduced with a MOI of 100, incubated at 37 ºC for 48 h. Then conditioned medium was collected and subjected to the assay as per the manufacturer’s protocol. For the in vivo assays, the serum collected on experimental day 9 was analyzed by ELISA for circulating human IFNβ.

### Statistical analysis

Statistical analyses were performed using GraphPad Prism version 5.0 for Windows (GraphPad Software, La Jolla California USA) as cited in each figure legend. As described in our previous work^47^, statistical significance was calculated as cited in each figure legend and were considered significant when *p* < 0.05. As described in Tamura et al. (2019), each test was chosen since we propose that the data meet the assumptions of the test. In vitro experiments were performed on at least three independent occasions using technical replicates as indicated in each figure legend*. *In vivo assays were performed with sample sizes as indicated in the figure legends.

## Supplementary information

Supplementary information.
