# Supplementary material for: Response of human melanoma cell lines to interferon-beta gene transfer mediated by a modified adenoviral vector
Source: Sci Rep. 2020 Oct 21;10:17893. doi: 10.1038/s41598-020-74826-y (PMC7578831; doi:10.1038/s41598-020-74826-y)
Supplement: Supplementary file 1 — Supplementary information. [file 41598_2020_74826_MOESM1_ESM.pdf]

Response of human melanoma cell lines to interferon-beta gene transfer mediated by a modified adenoviral vector

Taynah I.P. David[1,*], Otto L.D. Cerqueira[1,*], Marlous G. Lana[1], Ruan F.V. Medrano[1,2], Aline Hunger[1,3], Bryan E. Strauss[1,4]

*These authors contributed equally to this work.

1-Laboratório de Vetores Virais, Centro de Investigação Translacional em Oncologia, Instituto do Câncer do Estado de São Paulo, Faculdade de Medicina, Universidade de São Paulo, São Paulo, Brasil.

2-Present address: Department of Pathology & Immunology, Washington University School of Medicine, St. Louis, MO, USA.

3-Present address: Cristalia, Biotecnologia Unidade 1, Rodoviária SP 147, Itapira, SP, Brasil.

4: Corresponding author

Bryan E. Strauss

Laboratório de Vetores Virais

Centro de Investigação Translacional em Oncologia

Instituto do Câncer do Estado de São Paulo

Av. Dr. Arnaldo, 251, 8th floor

São Paulo, SP, Brasil

Postal code: 01246-000

Tel: +55(11)3893-3554

bstrauss@usp.br, bryan.strauss@hc.fm.usp.br

Running title: Human interferon-β gene transfer in melanoma

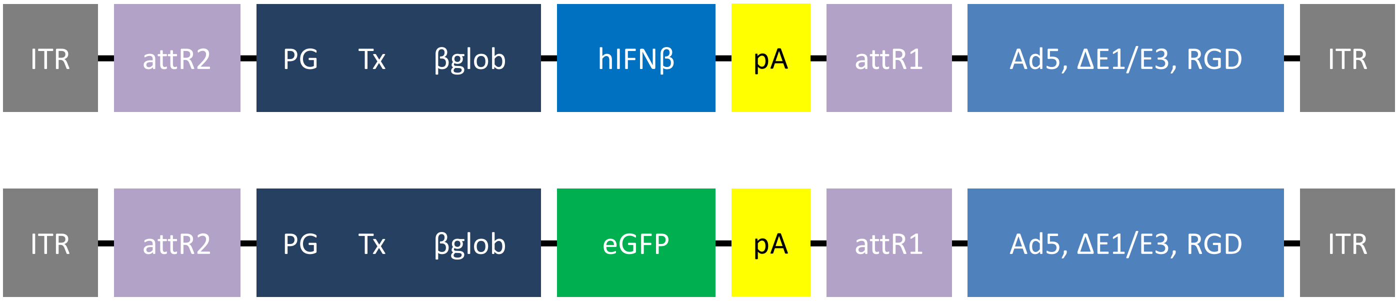

Figure S1. Schematic representation of the AdRGD-PG vectors. ITR, inverted terminal repeat; attR2, attR1, site specific recombination sites; PG Tx βglob, chimeric p53 responsive promoter, where PG represents the p53 responsive element, Tx, minimal promoter, and βglob, intron from rabbit β-globin gene; hIFNβ, human interferon-β cDNA; eGFP, enhanced green fluorescent protein cDNA; pA, polyadenylation site, Ad5, serotype 5 adenovirus backbone, ΔE1/E3, deletion of the E1 and E3 genes; RGD, tripeptide modification of the H1 loop of the knob protein.

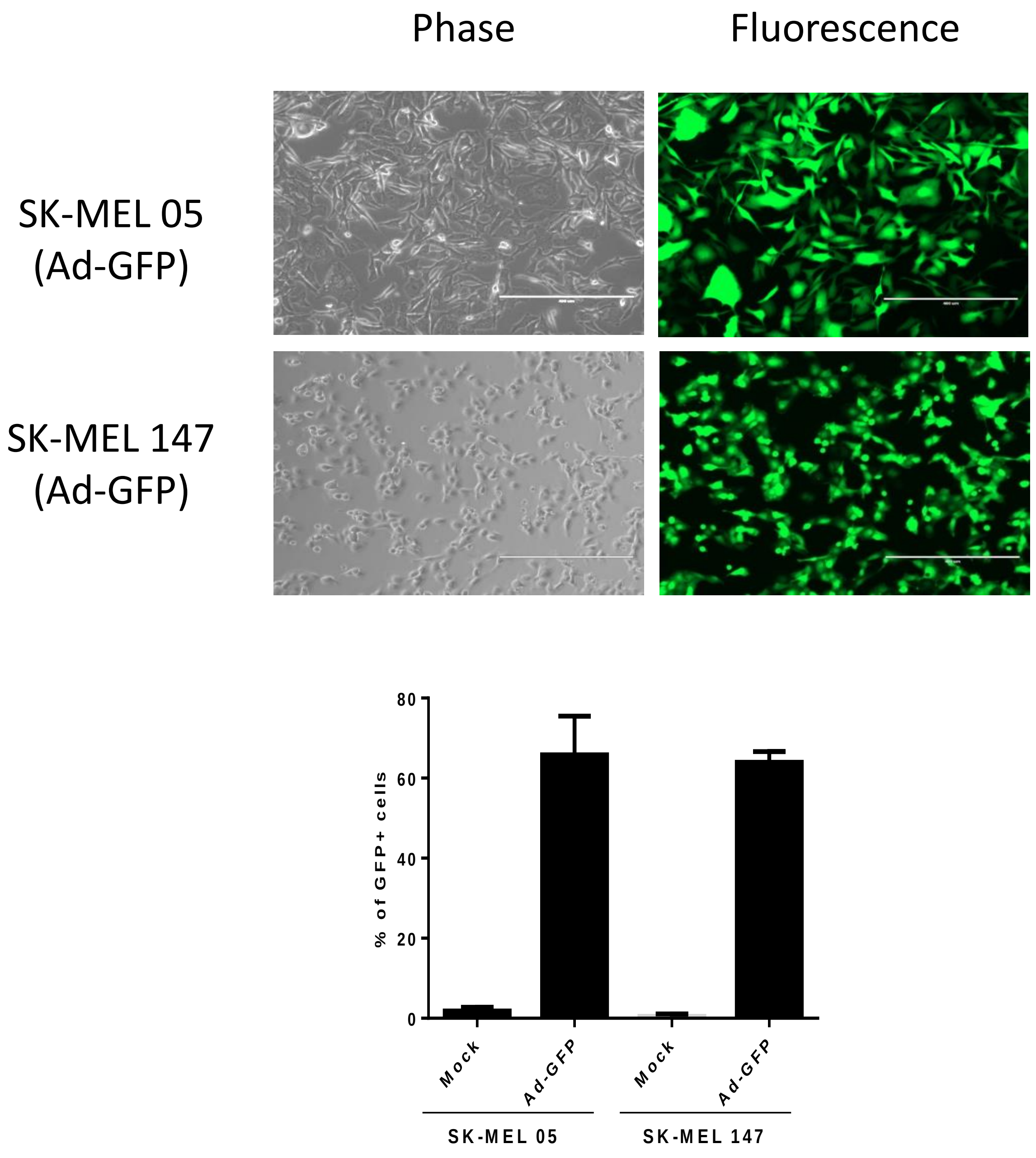

Figure S2. Validation of eGFP expression. Melanoma cell lines SK-MEL-05 and SK-MEL-147 were transduced with AdRGD-PG-eGFP (Ad-GFP, MOI of 100) or not (Mock) and, the following day, eGFP was observed by fluorescence microscopy (EVOS FL, Thermo Fisher Scientific) or flow cytometry (Attune™, Thermo Fisher Scientific).

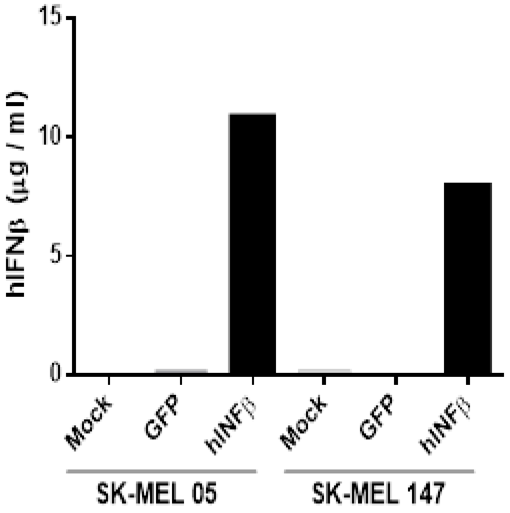

Figure S3. **Validation of hIFNβ expression.** The presence of hIFNβ was assessed by ELISA (VeriKine human interferon beta ELISA kit, PBL Assay Science, Piscataway, NJ, USA) in the supernatant of human melanoma cell lines SK-MEL-05 and SK-MEL-147 48 hours after transduction (MOI of 100) with AdRGD-PG-hIFNβ (hIFNβ), AdRGD-PG-eGFP (GFP) or a non-treated control (Mock).

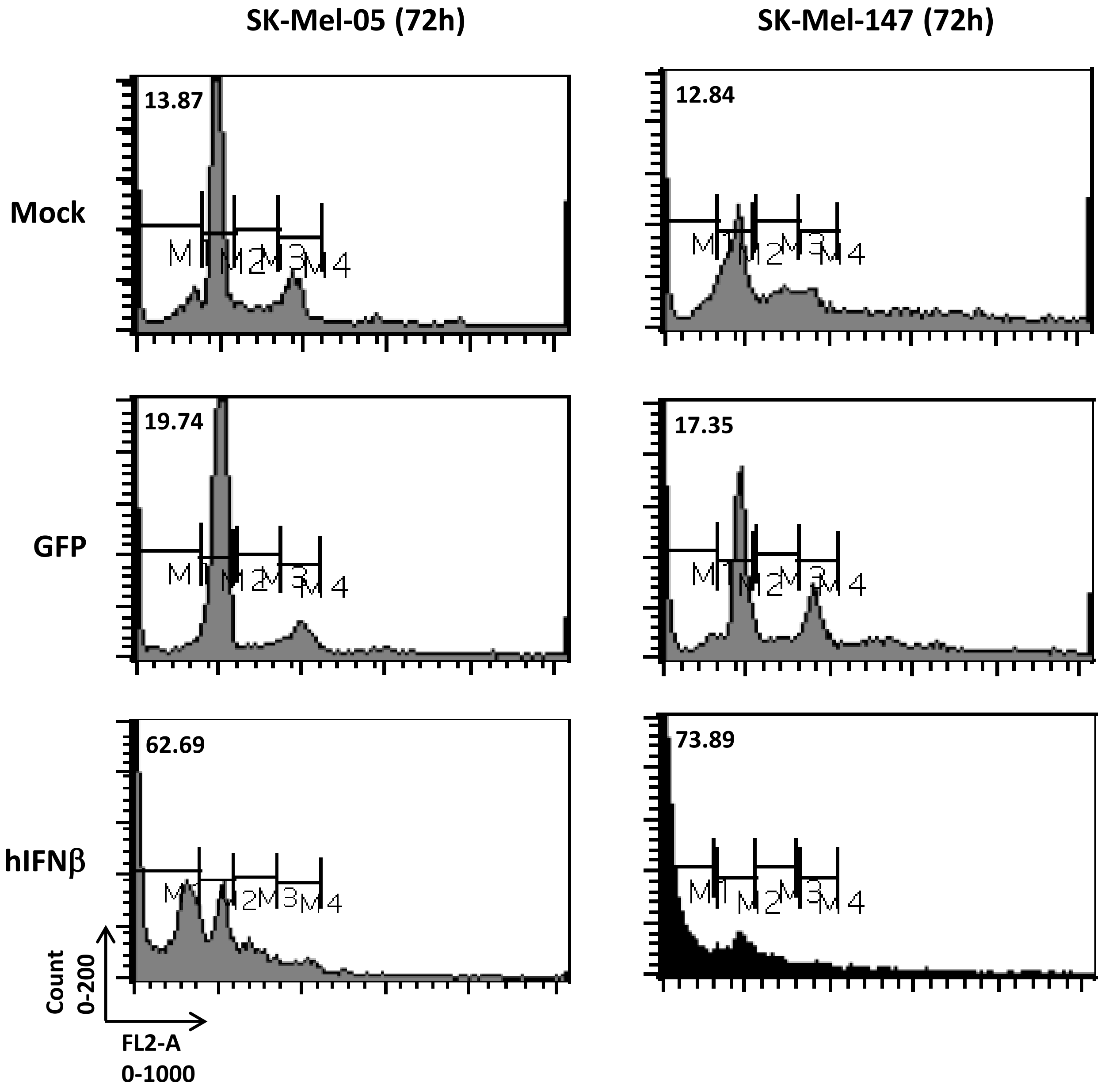

Figure S4. **Representative flow cytometry data showing accumulation of hypodiploid cells upon treatment.** As per Figure 1 of the main text, human melanoma cell lines SK-MEL-05 and SK-MEL-147 were transduced (MOI of 100) with AdRGD-PG-hIFNβ (IFNβ), AdRGD-PG-eGFP (GFP) or a non-treated control (Mock). After 72 hours incubation, the cells were harvested, fixed with 70% ethanol and treated with RNAse plus propidium iodide. The value shown represents the M1 region (hypodiploid/sub G1) cells.

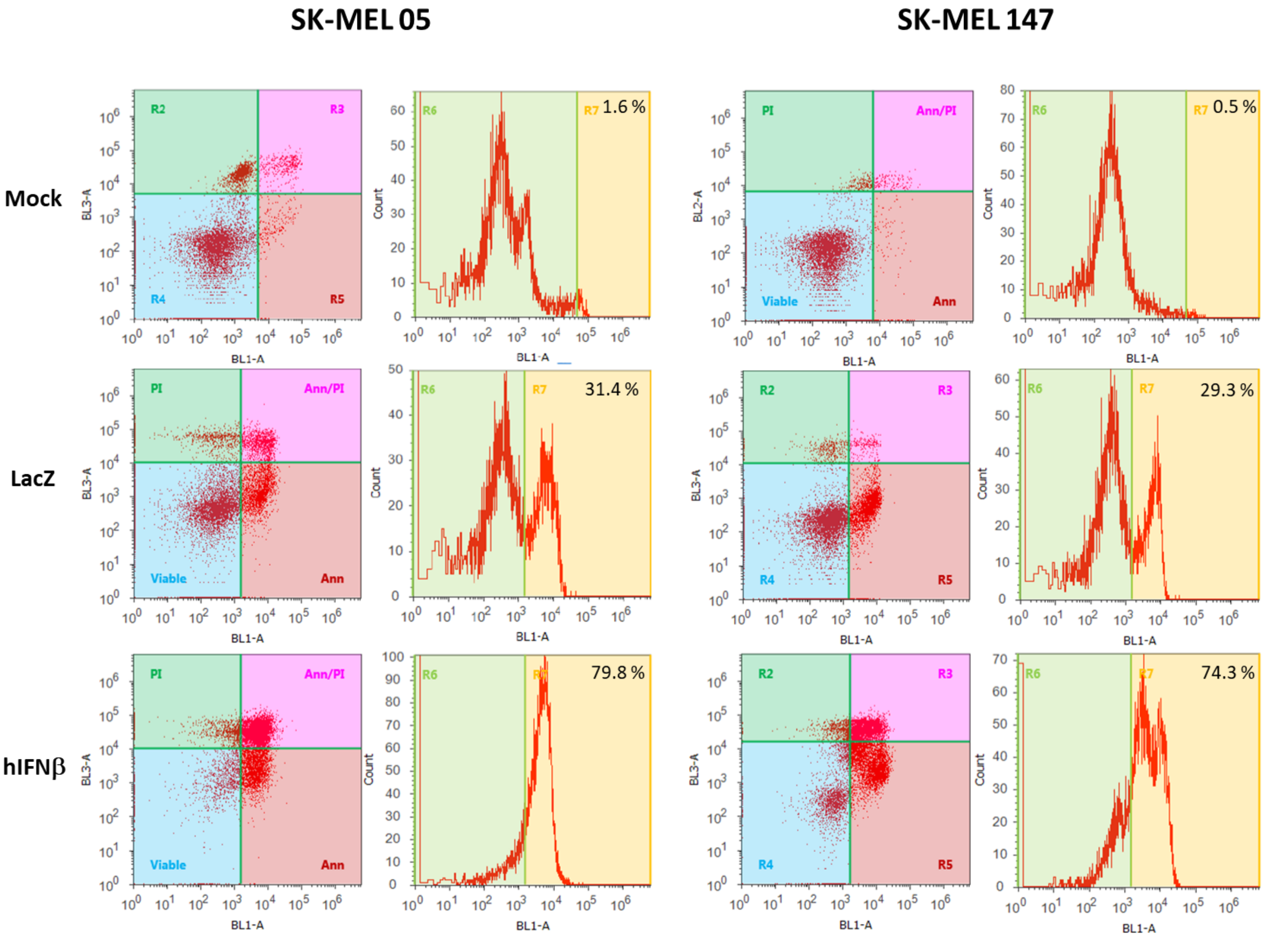

Figure S5. **Representative flow cytometry data showing annexinV staining.** As per Figure 2 of the main text, human melanoma cell lines SK-MEL-05 and SK-MEL-147 were transduced (MOI of 100) with AdRGD-PG-hIFNβ (hIFNβ), AdRGD-CMV-LacZ (LacZ, used here to avoid interference in the BS1-A channel) or a non-treated control (Mock). After 72 hours incubation, the cells were harvested and immediately stained with annexinV-FITC/propidium iodide before flow cytometry. BL1-A channel detects the signal from FITC while BL3-A reveals PI staining. The value shown represents the R1 region (Ann/PI + Ann).

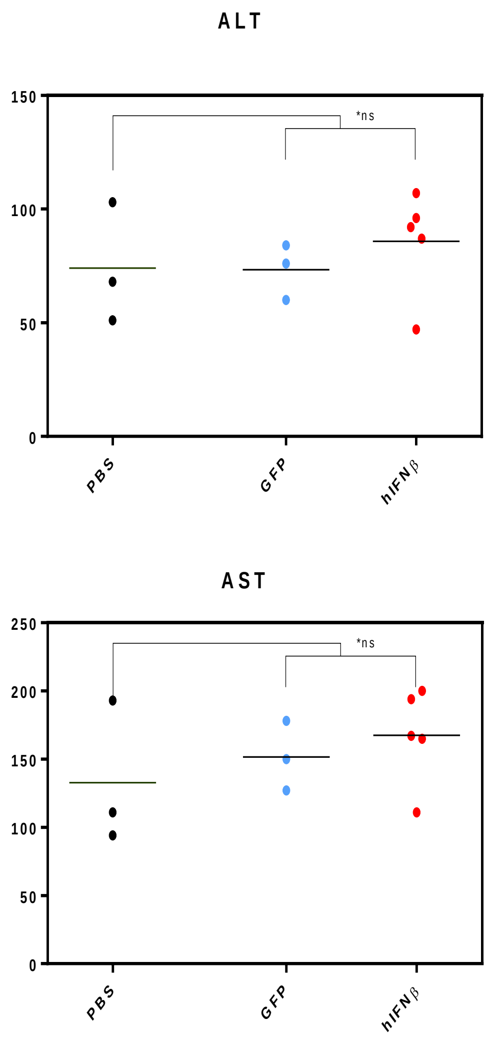

Figure S6. ***In situ* gene therapy was not associated with hepatic toxicity.** Animals treated as per Figure 4 of the main text were evaluated for hepatic toxicity by quantification of serum levels of aspartate aminotransferase (AST) and alanine aminotransferase (ALT). There were no significant changes in circulating ALT and AST levels in any of the mice.
